# Involvement of Endoplasmic Reticulum Stress in Palmitate-induced Apoptosis in HepG2 Cells

**DOI:** 10.5487/TR.2008.24.2.129

**Published:** 2008-06-01

**Authors:** Hyang-Ki Cho, Jin-young Lee, Yu-mi Jang, Young Hye Kwon

**Affiliations:** Department of Food and Nutrition, Research Institute of Human Ecology, Seoul National University, Seoul, 151-742 Korea

**Keywords:** Palmitate, ER stress, Lipotoxicity, Apoptosis, Chemical chaperone, HepG2 cells

## Abstract

The results of recent studies indicate that high levels of free fatty acids (FFAs) and adipokines may be the main causes of non-alcoholic liver disease; however, the molecular mechanism that links FFAs to lipotoxicity remains unclear. In the present study, we treated HepG2 cells with FFA (either palmitate or oleate) to investigate the mechanisms involved in lipotoxicity in the liver cells. We also treated cells with palmitate in the presence of a chemical chaperone, 4-phenylbutyric acid (PBA), to confirm the involvement of ER stress in lipotoxicity. Palmitate significantly induced cytotoxicity in dose- and time-dependent manners. Apoptosis was also significantly induced by palmitate as measured by caspase-3 activity and DAPI staining. Palmitate led to increased expressions of the spliced form of X-box-protein (Xbp)-1 mRNA and C/EBP homologous transcription factor (CHOP) protein, suggesting activation of the unfolded-protein response. PBA co-incubation significantly attenuated apoptosis induced by palmitate. The above data demonstrate that high levels of palmitate induce apoptosis via the mediation of ER stress in the liver cells and that chemical chaperones act to modulate ER stress and accompanying apoptosis.

## INTRODUCTION

Obesity can be considered as a chronic low-grade inflammatory state with adipose tissue secreting multiple cytokines, hormones, and free fatty acids (FFAs) that have wide-ranging metabolic effects (Dandona *et al.*, 2004; Hotamisligil, 2006). High FFA concentrations trigger apoptosis and insulin resistance of many tissues and at the core of this syndrome is the dysregulation of lipid metabolism (Hotamisligil, 2006; Kim, 2005). Nonalcoholic fatty liver disease (NAFLD) is now considered as the hepatic manifestation of the metabolic syndrome, and is present in ~80% of the type 2 diabetes (Moscatiello *et al.*, 2007). Previous studies have showed lipotoxicity induced by saturated fatty acids in several tissues and cells (Karaskov *et al.*, 2006; Wei *et al.*, 2006).

Several studies have suggested that endoplasmic reticulum (ER) stress is involved in the development of metabolic disease (Nakatani *et al.*, 2005; Ozcan *et al.*, 2004). The ER has been suggested as a site for the sensing of metabolic stress and the translation of that stress into inflammatory responses and cytotoxicity (Hotamisligil, 2006; Hwang *et al.*, 2002; Lee *et al.*, 2004). An increase in proteins requiring unfolding or changes in the ER environment can elicit the unfolded protein response (UPR), a mechanism that counteracts ER stress (Harding and Ron, 2002). If the counter-regulatory mechanisms such as elevating the ER chaperone or degrading unfolded proteins cannot compensate for the imposed ER stress, the pro-apoptotic transcription factor, C/EBP homologous transcription factor (CHOP), and other components of the apoptotic machinery are activated (Boyce and Yuan, 2006).

Groups of small molecules such as sodium 4-phenylbutyrate (PBA), dimethylsulfoxide, glycerol, and trimethylamine N-oxide have been shown to act as chemical chaperones by improving the misfolding and mislocalization of proteins such as a1-antitrypsin, prion proteins, aquaporin-2, cystic fibrosis transmembrane conductance regulator, and Parkin-associated endothelin receptor-like receptor (Bernier *et al.*, 2004; Kubota *et al.*, 2006;

Welch and Brown, 1996). Given the possible relation between ER stress and cytotoxicity, it is anticipated that chemical chaperones can inhibit ER stress and cell death in sequence.

FFA-involved apoptosis in the pancreatic β-cells (Karaskov *et al.*, 2006; Kharroubi *et al.*, 2004) and hepatocytes (Wei *et al.*, 2006) have been proposed in several studies, although the molecular mechanism by which FFA causes apoptosis is not fully understood. Therefore, in the current study we investigated the associated mechanism in an obesity-related liver disease model derived from high levels of FFA. We treated HepG2 cell lines with either palmitate or oleate, and found that palmitate treatment results in hepatic ER stress along with apoptosis.

## MATERIAL AND METHODS

***HepG2 cell culture and treatment.*** HepG2 human hepatocarcinoma cell line was obtained from ATCC (USA) and cultured in DMEM (5.6 mM L-glucose) supplemented with 10% fetal bovine serum (FBS), 1% penicillin-streptomycin at 37°C/5% $CO_2$. For FFA treatment, FFA solutions were prepared as described (Karaskov *et al.*, 2006) and the solution was diluted 1 : 5 in DMEM without FBS to a final concentration of 1 mM FFA/1% BSA. Chemical chaperones were pre-treated for 2 h after overnight serum deprivation and co-treated with FFA.

***Cell viability assay.*** Cell viability was measured using a 3-(4,5-dimethylthiazol-2-yl)-2,5-diphenyltetrazolium bromide (MTT) assay (Carmichael *et al.*, 1987). At the end of the incubation with FFA, cells were washed with PBS and the incubated in phenol-free DMEM with MTT (5 mg/ml PBS). Cells were incubated with for 3 h at 37°C in a humidified atmosphere of 5% $CO_2$. Then the medium was removed and the cells were incubated for 15 min with isopropanol. The absorbance of the MTT formazan was determined at 570 nm using a microplate reader (Bio-Rad, USA). In some experiments, the Cell Counting Kit-8 (Dojindo, Japan) was used to measure viable cell numbers as described by the manufacturer. Briefly, cells were suspended in 100 μl culture medium and inoculated in each well of 96-well plate at the density of $0.8 \times 10^4$ cells/well. After treatment, the cells were incubated with 10 μl of the CCK-8 solution for 4 h at 37°C. The absorbance was determined at 450 nm using a microplate reader.

***Determination of caspase-3 activity.*** Caspase-3 activity was measured by Caspase-3 colorimetric assay kit (MBL, USA) as described by the manufacturer. Absorbance was measured using a microplate reader (Bio-Rad, USA) at 405 nm and protein concentration was determined using the protein assay reagent (Bio-Rad, USA).

***DAPI staining.*** To detect apoptotic body, hepatocytes were stained with 4'-6-diamidino-2-phenylindole (DAPI). Briefly, cells were plated at 4-well chamber slides, which were coated with poly-lysine. After treatment with FFA for 6 h, medium was removed and cells were fixed with 4% paraformaldehyde and fixed cells were stored with 70% ethanol at -20°C until analysis. For staining, cells were incubated with 1 μg/ml DAPI for 10 min at room temperature, were washed twice with PBS, and then were photographed.

***Determination of expression of ER stress genes using semiquantitative RT-PCR.*** Total cellular RNA was isolated using Trizol Reagent (Life Technologies, USA) and cDNA was synthesized using 4 μg of total RNA with the Superscript®II first-strand synthesis system for RT-PCR (Life Technologies, USA). For amplification of cDNA, primers for Xbp-1 (upstream, AAACAGAGTAGCAGCTCAGACTGC; downstream, TCCTTCTGGGTAGACCTCTGGGAG) were used. Amplified products were further digested by PstI to check whether a PstI restriction site was lost after IRE1-mediated splicing of mRNA. Expression of β-actin was examined as an internal control (upstream, GTTTGAGACCTTCAACACCCC; downstream, GTGGCCATCTCCTGCTCGAAGTC). For each combination of primers, the kinetics of PCR amplification was studied, the number of cycles corresponding to plateau was determined, and PCR was performed within the exponential range. Amplified products were separated on an agarose gel and visualized with ethidium bromide staining.

***Cell extract preparation and immunoblotting.*** After treatment with FFAs, cells were lysed in the cold buffer containing 50 mM Hepes-KOH, pH 7.5, 150 mM NaCl, 1 mM NaF, 10% glycerol, 1 mM EDTA, 2.5 mM EGTA, 10 mM β-lycerophosphate, 0.1 mM $Na_3VO_4$, 1 mM DTT, 0.1% Tween-20 and 0.2 mM PMSF. For immunoblotting, equal amounts of total protein were resolved by SDS-PAGE and transferred onto Immobilon-P membrane (Milipore, USA). All membranes were stained with Ponceau S to confirm equal loading and transfer of protein. Following blocking with either 5% non-fat milk or 5% BSA, membranes were probed with specific primary antibodies and subsequently incubated with HRP-linked secondary antibodies for chemiluminescent detection (Pierce, USA). Densitometric analysis of western blots was performed and the Quantity one 4.3.1 program (Bio-Rad, USA). Primary antibodies were obtained as follows; α-spectrin (Chemicon, USA), CHOP (Santa Cruz biotechnology, USA), and β-actin (Sigma, USA).

***Statistical analysis.*** The data were analyzed using SAS software. For all experiments, either t-test or one-way ANOVA followed by Duncan's multiple range test was employed to assess the statistical significance. Data were expressed as mean ± SEM and statistical significant difference was considered to be present at $p < 0.05$.

## RESULTS

***Palmitate induces cytotoxicity of HepG2 cells.*** We treated HepG2 cells with either palmitate or oleate, to investigate the effect of elevated FFA levels on the liver. The venous blood concentration of FFAs is known to vary widely (~0.25-3.0 mM) and is chronically higher in individuals with obesity (Hamilton and Kamp, 1999). And local concentrations of FFAs in the capillaries is likely to be higher and to vary even more than that revealed by an analysis of venous blood (Hamilton and Kamp, 1999). Palmitate-induced cell death occurred in a time-dependent manner when HepG2 cells were treated with palmitate for various time intervals without overnight serum deprivation (Fig. 1A). Significant cell death compared to control (6 h) was apparent after 6 h of treatment with 1 mM palmitate. There was no significant change in cytotoxicity in cells treated with oleate. When cells were treated with various concentrations of palmitate (0~0.5 mM) for 12 h, relative cell viability was significantly decreased in a dose-dependent manner (Fig. 1B); however, there was no significant difference in the extent of cell toxicity by palmitate treatment at concentrations between 0.5 and 2 mM. Overnight FBS deprivation induced higher cytotoxicity compared to the previous data in Fig. 1A.

***Palmitate induces apoptosis of HepG2 cells.*** Based on our observation that palmitate acted to induce cytotoxicity, we found that HepG2 cells died in an apoptotic manner, as deduced from the results of DAPI staining and caspase-3 activity. After 6 h of 1 mM palmitate treatment without overnight serum deprivation, caspase-3 activity had significantly increased, whereas 1 mM oleate had no significant effect (Fig. 2A). After 6 h of 1 mM palmitate treatment after overnight serum deprivation, we were able to clearly observe the apoptotic bodies; in contrast, very few condensed and fragmented nuclei were observed in the control and 1 mM oleate-treated cells (Fig. 1D). We also observed that palmitate induced apoptosis of HepG2 cells, judging from the results for the cleavage of α-spectrin. The production of spectrin breakdown products (SBDP) is a well-recognized marker for the apoptosis induced by calpain and caspase-3 (Zhang *et al.*, 2006). We clearly observed an increase in SBDP when the cells were treated with palmitate for 6 h (Fig. 2C).

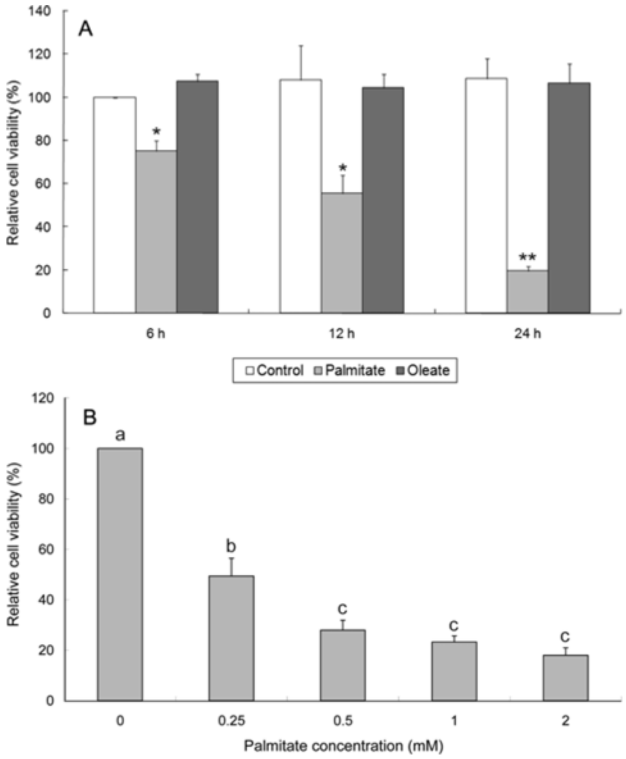

**Fig. 1.** Palmitate induces cytotoxicity of HepG2 cells. (A) Time-dependent effect of palmitate on cell viability was measured by MTT assay. Cells were treated with 1% BSA, 1 mM palmitate/1% BSA, or 1 mM oleate/1% BSA in media without serum for the times indicated. *$p < 0.05$ and **$p < 0.01$ compared with control in the same set (t-test). (B) Dose-dependent effect of palmitate on cell viability was measured by MTT assay. Cells were treated with various concentrations of palmitate/1% BSA in media without serum for 12 h. Bars not followed by the same superscript letter are significantly different ($p < 0.05$) by one-way ANOVA.

***Palmitate induces ER stress in HepG2 cells.*** Cellular markers of ER stress include splicing of Xbp-1 and up-regulation of CHOP (Kaufman, 2002). To determine whether FFA-induced cell death occurs as a result of ER stress, we examined whether palmitate can activate ER stress pathway signaling. Tunicamycin, which inhibits protein glycation, was used as a positive con-

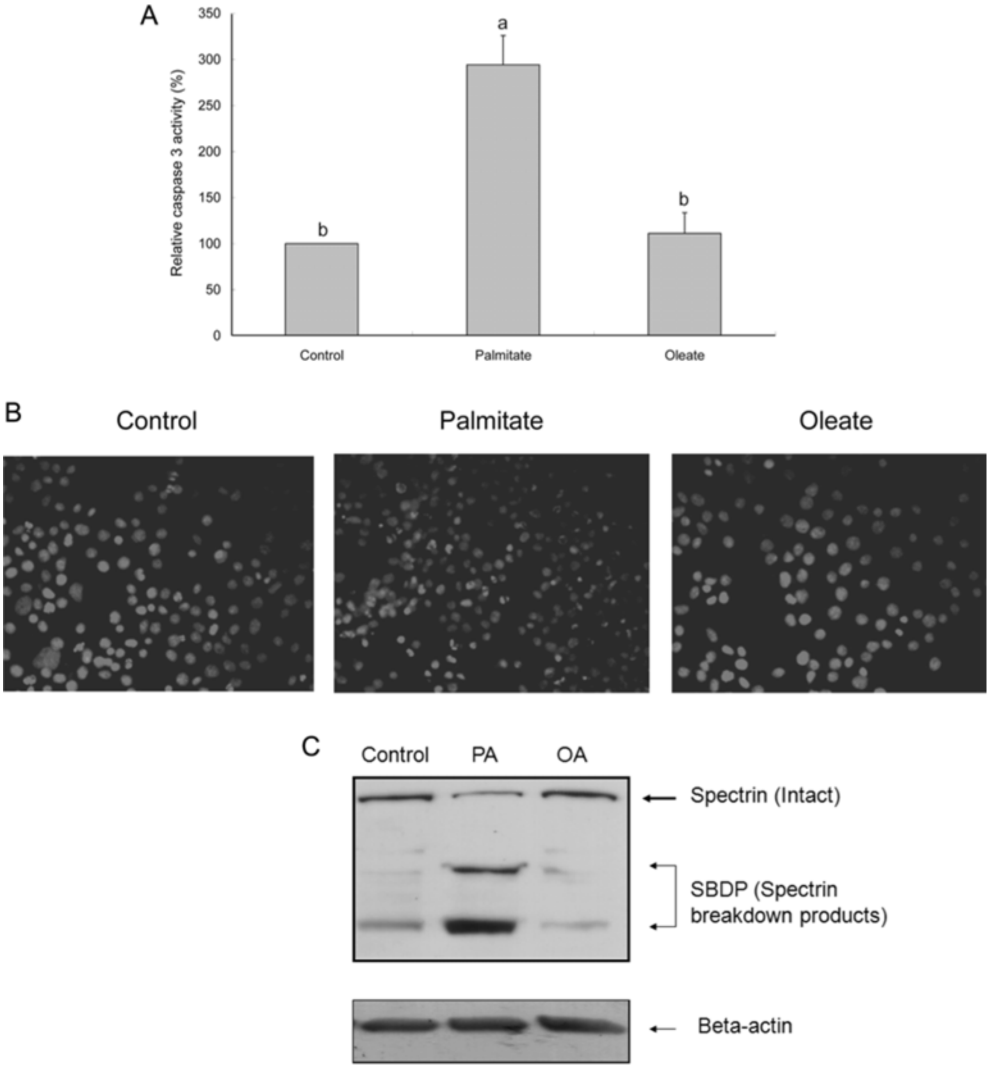

**Fig. 2.** Palmitate induces apoptosis of HepG2 cells. (A) Cell apoptosis by palmitate was measured by caspase-3 activity. Cells were treated with 1% BSA, 1 mM palmitate/1% BSA, or 1 mM oleate/1% BSA in media without serum for 6 h. Results are expressed as mean ± SEM (n = 3-4). Bars not followed by the same superscript letter are significantly different ($p < 0.05$) by one-way ANOVA. (B) Cells were exposed to different treatments for 6 h, stained with DAPI, and visualized under fluorescein UV optics. (C) Representative $\alpha$-spectrin and $\beta$-actin immunoblots from cells treated with each treatment for 6 h.

trol of ER stress. As shown in Fig. 3A, ER stress-induced splicing of Xbp-1 mRNA was markedly increased by 6 h of 1 mM palmitate treatment, but not by oleate treatment. In the state of persistent ER stress, the inositol-requiring enzyme 1 (IRE1) signaling pathway induces transcription and translation of proapoptotic factors such as CHOP (Kaufman, 2002). CHOP protein level was also significantly induced by palmitate, suggesting that overexpression of CHOP may promote cell death in palmitate-treated cells (Fig. 3B).

***Chemical chaperones attenuates palmitate-induced apoptosis.*** To determine the effect of chemical chaperones on cell viability, we treated cells with palmitate in the presence of a chemical chaperone, PBA. Cell viability was measured by a Cell Counting Kit-8 using cells treated with 0.5 mM plamitate in the presence of various concentrations of PBA for 6 h. As shown in Fig. 4A, cell viability was increased by dose-dependent manner in response to PBA treatment. We also measured caspase-3 activity to investigate whether chemical chaperone could inhibit apoptosis induced by palmitate. Caspase-3 activity was significantly restored to the level of control by 5 mM PBA (Fig. 4B). Overnight FBS deprivation induced higher caspase-3 activity compared to the previous data in Fig. 1C.

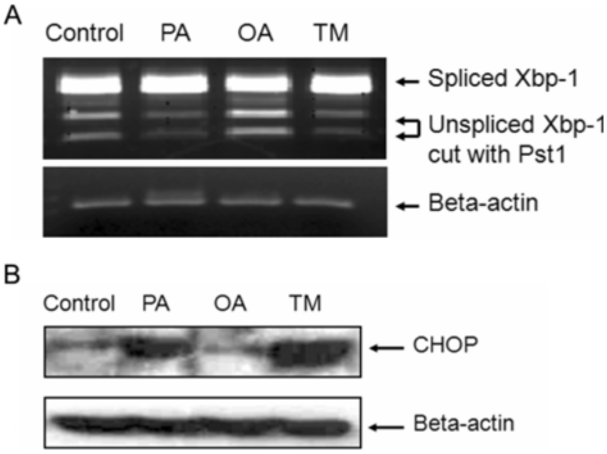

**Fig. 3.** Palmitate induces ER stress in HepG2 cells. (A) mRNA expressions of spliced and unspliced forms of Xbp-1 were measured by RT-PCR. (B) Protein expression of CHOP was measured by immunoblotting. To confirm the equal loading of the lysates, the membrane was reprobed with anti-β-actin antibody. Cells were treated with 1% BSA, 1 mM palmitate/1% BSA, or 1 mM oleate/1% BSA in media without serum for 6 h. Tunicamycin (TM) (5 μg/ml) was used as a positive control. Results are representative of 3 independent experiments.

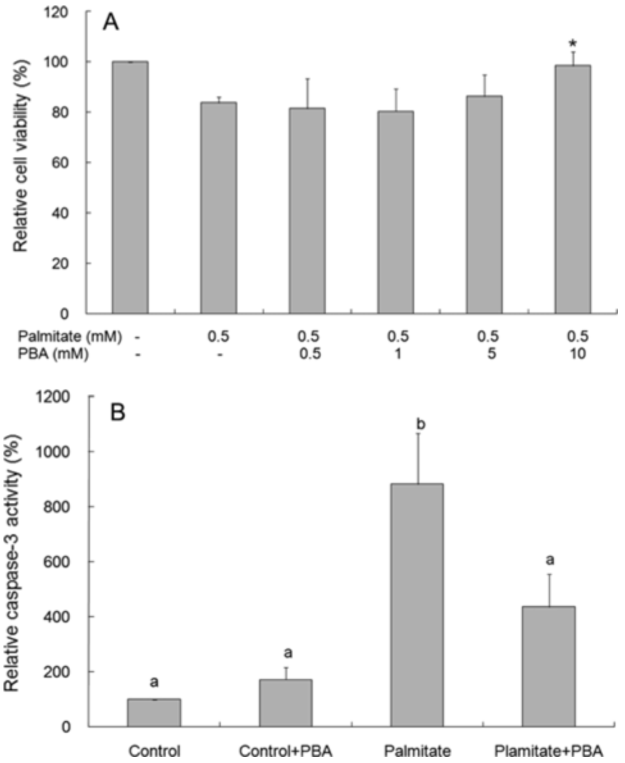

**Fig. 4.** Chemical chaperones attenuate palmitate-induced apoptosis of HepG2 cells. (A) Cell viability was measured by Cell Counting Kit-8. Cells were treated with 0.5% BSA or 0.5 mM palmitate/0.5% BSA in media without serum for 6 h. Results are expressed as mean ± SEM (n = 3). $*p < 0.05$ compared with palmitate-treated cells (t-test). (B) Cell apoptosis was measured by caspase-3. Cells were treated with 1% BSA or 1 mM palmitate/1% BSA in media without serum for 6 h. Results are expressed as mean ± SEM (n = 3). Bars not followed by the same superscript letter are significantly different ($p < 0.05$) by one-way ANOVA.

## DISCUSSION

Here, we show that palmitate, induced ER stress-mediated apoptosis in HepG2 cells. Lipotoxicity induced by a single species of fatty acid in the transformed cells may be considered too simple model to explain the metabolic changes associated with obesity (Moffitt *et al.*, 2005), because palmitate is found in the blood in combination with some other fatty acids, mostly oleate, and the co-treatment with unsaturated fatty acids attenuates palmitate-induced apoptosis (Guo *et al.*, 2007; Mishra and Simonson, 2005). However, the present study can be applied to explain the molecular mechanism involved in the ER stress-mediated lipotoxicity, which is known to be induced in high fat diet-mediated and gene-related obesity models. HepG2 cells retain many biochemical and morphological properties of hepatocytes including synthesizing many liver specific proteins as well as primary bile acids (Javitt, 1990). And the recent study reported proinflammatory effects of palmitate in HepG2 cells as well as in primary rat and human hepatocytes (Joshi-Barve *et al.*, 2007).

Several possible mechanisms exist which palmitate acts to induce ER stress. Previous studies suggested ceramide, a bioactive sphingolipid derived from long-chain saturated fatty acid, as a modulator of palmitate-mediated cell toxicity and insulin resistance (Holland *et al.*, 2007; Summers, 2006); however, Wei *et al.* (2006) reported that palmitate-induced ER stress was caused independently by ceramide accumulation in liver cells. Palmitate *per se* may contribute to ER stress by modulating the integrity of ER membrane. The recent study demonstrated that incorporation of palmitate in microsomal membranes of CHO cells resulted in dramatic dilatation of the ER and redistribution of protein-folding chaperones to the cytosol within 5 h, indicating compromised ER membrane integrity by palmitate (Borradaile *et al.*, 2006).

The present study showed that PBA inhibited apoptosis induced by palmitate, thereby confirming the role of ER stress in lipotoxicity in the liver. Previous studies using human neuroblastoma cells (Kubota *et al.*, 2006) and human embryonic kidney cells (Yam *et al.*, 2007) also showed the antiapoptotic effect of PBA. Ozcan et al. (2006) showed that chemical chaperones, such as PBA and the taurine-conjugated derivative of ursodeoxycholic acid (TUDCA) reduced ER stress in Fao liver cells treated with tunicamycin. They also showed that chemical chaperones restored glucose homeostasis in a mouse model of type 2 diabetes. PBA has been approved by the U.S. Food and Drug Administration as a therapeutic agent in the treatment of urea cycle disorders by scavenging ammonia. In addition, a pharmacological activator of AMP-activated protein kinase (AMPK), such as AICAR, has been shown to protect cardiomyocytes against ER stress-mediated apoptosis (Terai *et al.*, 2005). Although the detailed protective pathways are far from being understood, AMPK-dependent phosphorylation of eukaryotic elongation factor-2 may play a role in cardioprotective signaling mechanisms during hypoxia by suppressing protein synthesis (Chan *et al.*, 2004), suggesting AMPK activator may be useful in protection of hepatocytes in palmitate-mediated apoptosis.

In conclusion, we have shown that palmitate induced an ER stress that exceeds the capacity of the UPR, resulting in apoptosis in the liver cells. We also demonstrated that chemical chaperones inhibited ER stress-mediated apoptosis by palmitate. Further studies are required to identify the nature of the ER perturbations elicited by palmitate or palmitate-derived metabolites.

## ACKNOWLEDGMENTS

H.-K. Cho, J.-Y. Lee, and Y.-M. Jang are supported by a second-stage BK21 Research Fellowship from the Ministry of Education and Human Resources Development.
